# Breaking Through the Limits: Nanomedicine at the Service of New Drug Combinations to Tackle Pancreatic Cancer

**DOI:** 10.1002/wnan.70067

**Published:** 2026-06-18

**Authors:** Valeria Bincoletto, Ilaria Andreana, Barbara Stella, Nazanine Modjtahedi, Silvia Arpicco, Giorgia Urbinati

**Affiliations:** ^1^ Department of Drug Science and Technology University of Turin Turin Italy; ^2^ Unité Physiopathologie et Génétique du Neurone et du Muscle Université Claude Bernard Lyon 1 Lyon France; ^3^ Université Paris Cité, CNRS, INSERM, UTCBS Paris France

**Keywords:** combinatory treatments, disulfiram, gemcitabine, liposomes, nanomedicine, pancreatic cancer

## Abstract

Pancreatic ductal adenocarcinoma (PDAC) is among the most aggressive cancers, with a poor prognosis due to late diagnosis and resistance to chemotherapy. Gemcitabine (GEM) monotherapy was the gold standard treatment for PDAC until the early 2010s, when two combinatorial therapies, FOLFIRINOX and GEM combined with Nab‐paclitaxel, showed the benefits of the multi‐drug approach and became the reference treatments for PDAC. Despite their undisputed efficacy, the overall survival of treated PDAC patients is very low, reaching approximately 12% at 5 years, and the side effects of these therapeutic protocols remain severe and not tolerated by all patients. Recent advances in understanding PDAC biology have led to new therapeutic strategies, including new drug combinations and nanomedicine. This review summarizes background information about past and present PDAC therapeutic regimens with their benefits and the drawbacks including the appearance of treatment resistance and focuses on two potential strategies to counteract the limitations of the actual therapies. First, we highlight the interest of combining disulfiram, a repurposed anti‐alcoholism drug, with GEM, based on evidence of synergism between the two molecules. We then emphasize the use of drug delivery nanosystems for their ability to improve drug stability, targeting and to potentially overcome resistance and reduce side effects. Finally, we discuss the combination of multi‐drug therapies and nanomedicine through the design of apposite drug delivery nanocarriers capable of encapsulating more than one drug and ensuring sustained release. This all‐in‐one approach should be promising for more effective therapies of this challenging disease.

This article is categorized under:

Therapeutic Approaches and Drug Discovery > Nanomedicine for Oncologic Disease
Biology‐Inspired Nanomaterials > Lipid‐Based Structures

## Graphical abstract

Pancreatic cancer is a highly aggressive tumor that responds poorly to current therapies. New multi‐drug combinations and/or the use of drug delivery nanosystems may lead to the development of more effective and better‐tolerated treatments for this challenging disease.

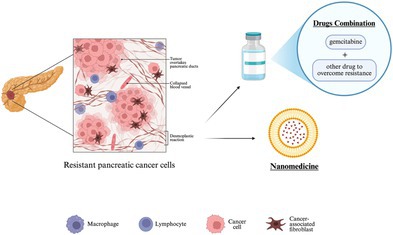

## 1 Introduction

Pancreatic ductal adenocarcinoma (PDAC) is a highly aggressive cancer that remains a significant clinical challenge due to its late diagnosis and resistance to chemotherapy. Approximately 90% of PDAC cases are diagnosed at an advanced stage, with unresectable disease and after metastases have occurred. This late detection has a significant impact on the efficacy of the anticancer therapies and therefore on the survival rate of the patients, which is nearly 12% at 5 years (Halbrook et al. 2023). The median survival time for patients drops to less than 1 year when the disease is diagnosed at stage IV, regardless of the best available chemotherapy protocols (Springfeld et al. 2019). Consequently, PDAC exhibits one of the poorest survival rates among all types of cancer (Hu and O'Reilly 2024), and unfortunately “old molecules” such as gemcitabine (GEM), a drug approved in 1996, remain the standard of care for this disease, particularly for patients with fragile overall health, who cannot support more effective but less tolerated therapeutic options (Kung and Yu 2023).

Nevertheless, over the past decade, new avenues for potential therapies have been discovered as a result of a better understanding of the biological mechanisms underlying PDAC. However, when it comes to patients diagnosed at later stages, there is still an urgent need for more effective treatments for the metastatic and chemotherapy‐resistant diseases (Wood et al. 2022). In addition, pre‐existing or acquired resistance can limit the clinical benefit of even the most advanced therapies.

Overcoming resistance and providing new treatment options can be achieved by applying strategies such as the combination of cancer drugs and the nanovectorization of chemotherapeutics, namely nanomedicine. Combinations of targeted anticancer agents have the potential to improve response to existing drugs and expand treatment options. For example, FOLFIRINOX combination therapy [oxaliplatin, irinotecan, leucovorin calcium, 5‐fluorouracil (5‐FU)] has shown promising results in treating patients with PDAC at stage IV, extending the overall survival from 6.6 months, when GEM was administered as monotherapy, to 11.3 months when the FOLFIRINOX protocol was introduced (Conroy et al. 2011; Gourgou‐Bourgade et al. 2013). When it comes to identifying new drug combinations, repurposed therapeutics may also attract much attention, in particular if their molecular mechanism of action is well known. In this context, disulfiram (DSF), a drug approved for the treatment of alcohol dependency, has recently been repositioned to exploit its anti‐cancer properties (Xu, Lu, et al. 2021).

Another fruitful strategy to overcome resistance is the loading of therapeutics into nanodelivery systems, which act as carriers and modify the solubility, distribution, mechanisms of cellular uptake and, ultimately, the efficacy of the active principles. This leads to improved treatments and, if the nanodelivery system is properly modified to recognize specific cell types, the result is a more selective drug delivery and a reduction in side effects. The two approaches, combining drugs and using nanomedicine, can be successfully merged, as in the case of the formulation of paclitaxel‐loaded albumin‐stabilized nanoparticle (Nab‐paclitaxel), which was combined with GEM in a randomized phase III trial. This treatment increased the overall survival compared to individual treatments (Von Hoff et al. 2013).

This review will explore how the combination of multi‐drug therapies and nanomedicine could help overcome resistance to traditional PDAC treatments.

## 2 GEM Treatment of PDAC and Occurrence of Resistance

Since 1996 and until a few years ago, GEM monotherapy was the first‐line treatment for patients with advanced PDAC, as clinical and survival benefits were observed compared to patients treated with 5‐FU (Burris et al. 1997; Pandit and Royzen 2022). For patients with compromised health, this molecule remains the standard of care.

### 2.1 GEM and Its Mechanism of Action

**FIGURE 1:**
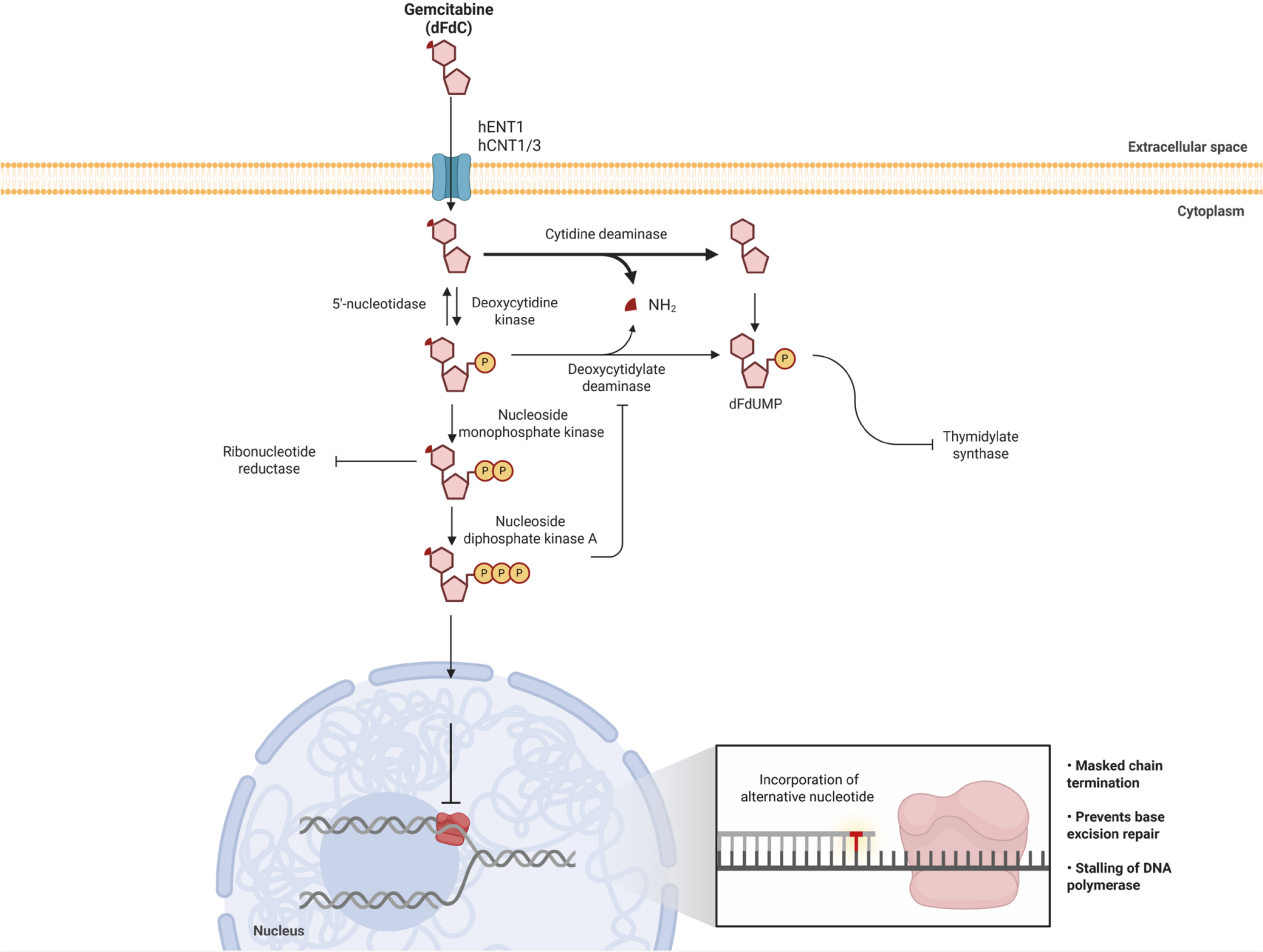
Mechanisms of GEM action (created with Biorender).

GEM is a synthetic nucleoside analogue widely used as an antimetabolite antineoplastic drug. The molecular targets of GEM are intracellular and therefore crossing the plasma membrane is necessary to obtain its pharmacological activity. GEM uses complex transport systems to penetrate the cell (Figure 1). These systems consist of several nucleoside transporter proteins (NTs). There are two major classes: the human equilibrative nucleoside transporters (hENTs) and the human concentrative nucleoside transporters (hCNTs) (Carter et al. 2021). The hENTs' family is composed of four members, the first three differ in nucleoside specificity while hENT4 has poor affinity for nucleosides and is mainly a monoamine transporter (e.g., dopamine, serotonin). The hCNTs' family is composed of three members, which are responsible for the unidirectional co‐transport of nucleosides and sodium (Na+) or protons (H+) into cells. The hENT1 is likely the main transporter of GEM (Gesto et al. 2012). After internalization, GEM is subsequently phosphorylated by deoxycytidine kinase (dCK) and nucleoside mono‐ and di‐phosphate kinases to become the active form of GEM: GEM triphosphate (20,20‐difluoro‐20‐deoxycytidine triphosphate, GEM‐TP) (Derissen and Beijnen 2020).

GEM‐TP acts mainly by competing with deoxycytidine triphosphate to interfere with DNA synthesis. GEM‐TP is incorporated into a single DNA strand by the DNA polymerase during replication. Once incorporated into DNA, GEM‐TP leads to premature chain termination after the insertion of another nucleotide triphosphate (dNTP). This GEM‐TP position, known as “masked chain termination”, inhibits the removal of GEM‐TP by DNA repair enzymes, ultimately leading to single‐strand breakage (Beutel and Halbrook 2023) (Figure 1). Among all the other mechanisms of action of GEM, the active molecule could also be incorporated into RNA to limit RNA synthesis, inhibit topoisomerase I cleavage complexes by enhancing their stability, and thus be used to improve the efficacy of immunotherapies against cancers that are traditionally unresponsive. In addition, metabolites of GEM may also inhibit other metabolic enzymes, thereby indirectly increasing the relative cytotoxicity of GEM (Carter et al. 2021; Larson et al. 2024).

### 2.2 GEM and Its Mechanism of Resistance

Cancer therapy resistance can be classified as primary, when treatment is ineffective from the outset, or acquired, when resistance develops after an initial response. Resistance may arise from changes within cancer cells (cell‐intrinsic mechanisms) or the tumor microenvironment (non‐cell autonomous resistance). Both types pose significant challenges in treating PDAC. Cancer cells can evade treatment by reducing GEM uptake, enhancing detoxification, or undergoing epigenetic changes that alter their state, making them less responsive to therapy (Wood et al. 2022).

GEM resistance in PDAC is driven by several mechanisms. The role of nucleoside transporters like hENT1 is debated, with some studies linking low hENT1 expression to poor survival, while others find no survival impact (Carter et al. 2021; Xi et al. 2020). Resistance can also result from the downregulation of dCK, which activates GEM, with high dCK expression associated with better survival (Dash et al. 2024; Sebastiani et al. 2006). GEM must outcompete endogenous deoxycytidine, but increased ribonucleotide reductase (RR) activity, especially RRM1, can reduce its incorporation into DNA, contributing to resistance (Li, Xing, et al. 2024). Elevated glucose uptake and flux into the pentose phosphate pathway (PPP) also support resistance by enhancing nucleotide synthesis (Shimoni‐Sebag et al. 2024). Cytidine deaminase (CDA) mediated GEM detoxification, influenced by genetic polymorphisms, further reduces treatment efficacy, with high CDA activity being linked to poor outcomes and low activity associated with toxicity (Lumeau et al. 2024).

New mechanisms of resistance include p53 gene mutations, which are one of the most commonly mutated tumor suppressor genes (mutation rate of p53 in PDAC can be as high as 80%) (Tiwari et al. 2020). GEM treatment induces a cellular DNA damage‐response signaling cascade that involves the activation of several factors, including p53. Once activated, p53 induces cell cycle arrest, allowing and facilitating DNA repair. However, once mutated, p53 loses its cell growth inhibitory action and its “Guardian of genome” function; therefore, cancer cells can survive and carry even more mutations, increasing their aggressiveness. It is proposed that mutation of p53 increases resistance to GEM (Wu et al. 2020).

These findings underscore the variety of resistance mechanisms that limit the efficacy of GEM in PDAC and the need for alternative treatment options.

## 3 Current Combinatorial Treatments in Clinic

In 2011 and 2013, two large phase III clinical trials proved the clear benefit of two multi‐agent regimens: the FOLFIRINOX and GEM combined with Nab‐paclitaxel, compared with GEM monotherapy in terms of overall survival, which was significantly improved in both cases (Conroy et al. 2011; Von Hoff et al. 2013).

These two combinatorial therapies are now the two main first‐line options for patients with advanced PDAC; however, both treatments are associated with severe adverse effects and cannot be administered to all patients. Nevertheless, the major breakthrough of drug combination to treat PDAC and the necessity to find less toxic regimens have paved the way for the examination of new multi‐drug therapeutic options. GEM remains one of the most widely used drugs in combination therapies, as its side effects are far less severe than those of the above‐mentioned drugs (Robatel and Schenk 2022).

Indeed, in clinical trials, GEM is being combined with various classes of drugs, including other antimetabolites. In particular, the effects of combining GEM with S‐1 and capecitabine (5‐FU prodrug) have been studied (Eguchi et al. 2019; Palmer et al. 2025). Another widely used drug combination option is GEM administration together with kinase or farnesyltransferase inhibitors (e.g., erlotinib, afatinib, AL2846). Among them, tyrosine kinase inhibitors are the most frequently used. These combinations stop tumor progression and induce programmed cell death (Jo et al. 2022; Liu, Ji, et al. 2025; Zhang et al. 2024). Monoclonal antibodies have been combined with GEM in the treatment of pancreatic cancer to enhance its efficacy (Coveler et al. 2024; Li, Li, et al. 2024; Oberstein et al. 2024; Qin et al. 2023).

More recently, multi‐drug therapeutic options have focused on combining standard‐of‐care drugs with repurposed drugs whose mechanism of action and side effects are already known. The chapter below will address the combination of the old drug DSF with GEM and the potential interest of this combination for the treatment of PDAC.

## 4 Potential New Combinations With the Repurposed Drug: Disulfiram

Repurposing (or repositioning) drugs is a strategy for identifying new applications for existing drugs that offer advantages over developing entirely new drugs. The main advantages include a reduced risk of failure, a faster development time, and a lower cost, as the pre‐clinical testing and safety assessments have already been completed. Savings in the early stages of development can make repurposed drugs more cost‐effective, although regulatory and phase III costs can remain similar to those of new drugs (Pushpakom et al. 2019).

### 4.1 Disulfiram and Its Mechanism of Action

Disulfiram (DSF) is a drug candidate with significant potential for repositioning in cancer treatment; it is an anti‐alcohol drug approved by the US Food and Drug Administration in 1951 (Ekinci et al. 2019). It acts as a non‐selective and irreversible acetaldehyde dehydrogenase (ALDH‐1 and 2) inhibitor and causes acetaldehyde accumulation after alcohol consumption. This accumulation provokes several unpleasant reactions including tachycardia, nausea, flushing accompanied by headache and vomiting symptoms which should diminish the desire to consume alcohol (Lehner et al. 2024). Interestingly, the ability to reduce the expression of ALDH has also been described as an anticancer property as ALDH is a marker of cancer stem cells (CSCs) in a wide variety of tumors, and a key enzyme for the stemness of CSCs (Yang et al. 2019). Indeed, DSF was found to possess several anticancer properties (Bu et al. 2019; Hu et al. 2020; Loo et al. 2004) with excellent tumor‐selective toxicity (Iljin et al. 2009).

### 4.2 Disulfiram in Cancer Therapy

The mechanism by which DSF exerts its anti‐cancer effect has been investigated for many years and in various cancers (Ekinci et al. 2019), revealing its multi‐functional role. Li et al. identified the disruption of the ubiquitin‐proteasome system by DSF, which results in cancer cell death (Li 2020). Several studies suggest an anti‐proliferative effect linked to the production of reactive oxygen species (ROS), which are involved in apoptosis‐related cell death. Indeed, elevated ROS levels lead to molecular damage and oxidative stress (Liu et al. 2022). Furthermore, DSF can form disulfide bridges with the cysteines of enzymes or other proteins, inhibiting their function. One such protein is the glycoprotein P (P‐gp), an adenosine triphosphate efflux pump localized in the plasma membrane and capable of expelling a wide variety of drugs from the cell, thus conferring multi‐drug resistance to cancer cells. DSF, by inactivating the P‐gp, contributes to reducing the development of resistance to anticancer drugs (Loo et al. 2004).

### 4.3 Disulfiram for PDAC Treatment and Synergistic Effects When Combined With GEM in Preclinical and Clinical Trials

When the effects of DSF were studied on PDAC, Xu Y. et al. found that DSF suppressed the survival of pancreatic cancer cells after ionizing radiation exposure, both in vitro and in vivo. The results suggested that DSF may function as a radiosensitizer for pancreatic cancer, potentially by enhancing DNA damage, cell cycle arrest, and apoptosis (Xu, Lu, et al. 2021).

Furthermore, some studies have been focused on enhancing the synergistic antitumor effect of DSF by complexing it with copper (Cu) (Marengo et al. 2019). Yao Z. et al. observed that the DSF/Cu combination inhibited cell proliferation and induced apoptosis. In fact, DSF/Cu treatment induced autophagy by the upregulation of the transcription factor p8 and activation of the PI3K/mTOR pathway (Yao et al. 2022). Zhang X. et al. investigated the role of DSF/Cu in autophagy and apoptosis in pancreatic and breast cancer cells. DSF/Cu induced autophagy‐dependent apoptosis by activating the IRE1α‐XBP1 pathway, a key component of the unfolded protein response (UPR). These results suggest that the use of DSF/Cu, which induces ER stress and contributes to autophagy‐dependent cancer cell death, could lead to the development of new therapeutic strategies for pancreatic and breast cancer (Zhang et al. 2019).

DSF has also been combined with several other anticancer drugs, among them, GEM. Dalla Pozza et al. investigated the combination of GEM and DSF, with and without zinc (Zn), on pancreatic cancer cells and tumor growth in mice. Their findings revealed that the combination therapy resulted in enhanced cell death compared with drugs used individually, showing a link between cancer cell resistance to GEM and low levels of ROS. Combining GEM and DSF increased ROS levels, which was enhanced by Zn. In human pancreatic cancer models, the combination of GEM, DSF, and Zn exhibited robust antitumor effects. Indeed, this combination led to a substantial reduction in tumor mass, nearly halting tumor growth (Marengo et al. 2019).

Kim, S.K. et al. investigated the role of DSF in suppressing a pancreatic cancer cell subpopulation expressing high levels of ALDH and known for its high resistance to GEM. As DSF is an irreversible ALDH inhibitor, the reduction of ALDH levels induces apoptosis in cancer cells, preventing them from regaining their resistant properties. Combining DSF with low‐dose GEM has shown promising results in suppressing tumor growth in experimental models while sparing hematopoietic stem cells from significant damage (Kim et al. 2013).

Although the literature on the combination of DSF and GEM in pancreatic cancer is limited, the promising results (both for the efficacy of this combination in pancreatic cancer and its effectiveness in other types of tumors (Guo et al. 2010; Liu et al. 2021)) have allowed the development of two clinical trials.

Jatoi M. started a phase I clinical study in the USA that was conducted in patients with refractory solid tumors or metastatic pancreatic cancer to assess the optimal dose of DSF in combination with GEM and other chemotherapy. The trial was also evaluating whether DSF could reduce tumor‐induced muscle wasting and increase the sensitivity of tumor cells to chemotherapy. This trial aimed to determine the maximum tolerated dose (MTD) of DSF combined with GEM in patients with inoperable solid tumors. Among the secondary aims are the evaluation of side effects, toxicity profile, overall survival, and response rate. The study was recently closed due to a lack of funding (https://clinicaltrials.gov/search?cond=NCT02671890).

Jameson G. set up a phase II study to evaluate DSF and copper gluconate in patients with metastatic pancreatic cancer who have increased levels of the tumor biomarker carbohydrate antigen 19‐9 (CA‐19‐9) levels, despite treatment. Enrolled patients must have received at least 8 weeks of treatment with Nab‐paclitaxel plus GEM, FOLFIRINOX, or GEM alone and have increased levels of CA‐19‐9 without radiographic progression. The study had three treatment arms of five patients each, based on prior treatment. The study measured changes in plasma CA‐19‐9 level (at least 30%) from baseline and overall response rate. One patient was treated, but this patient was on study for 6 weeks and ended trial participation due to progressive disease. The study was closed due to the low subject enrolment at site (https://clinicaltrials.gov/search?cond=NCT03714555).

### 4.4 Limitation of GEM and DSF in Clinical Use

Despite the high efficacy of DSF in various cancer cells, its clinical effectiveness as a cancer treatment remains limited. This is due to its rapid metabolism and degradation by the liver and consequently its short half‐life, which prevents it from reaching and accumulating in the tumor.

Similarly, GEM faces limitations due to its rapid clearance, limited stability, and significant adverse effects. In addition, the cellular uptake of GEM depends on membrane transporters, and its activation requires intracellular phosphorylation. Consequently, its efficacy is closely related to the expression of the corresponding transporters and kinases, the reduction of which leads to the occurrence of resistance (Beutel and Halbrook 2023; Lowery and O'Reilly 2012).

To improve the therapeutic efficacy of DSF and GEM and to overcome their drawbacks, researchers have explored the use of advanced drug delivery systems in addition to the aforementioned drug combination strategy. These include nano‐encapsulation techniques to protect the functional groups and increase drug concentration at the tumor site. Section 5 describes the latest nanodelivery systems used to encapsulate GEM or DSF and the therapeutic benefits of this encapsulation. When the use of nanodelivery systems to protect the molecules joins the strategy of combinatorial treatments, consideration should be given to the use of co‐delivery systems for loading different molecules in the same nanovector. The combination of these two strategies may further enhance the synergistic effect and help overcome drug resistance (Section 5.3) (Lu et al. 2022).

## 5 Nanomedicine

### 5.1 Benefits of Using Nanomedicine to Treat PDAC

Surgical removal of tumors followed by systemic chemotherapy has constituted the primary treatment modality for PDAC for the past decade. However, factors such as late‐stage diagnosis, metastasis, and the dense tumor microenvironments (TMEs) observed in many cases impede the efficacy of this approach. PDAC TMEs are characterized by desmoplasia. This rigid barrier hinders the infiltration of immune cells and the penetration of therapeutic agents, contributing to multi‐drug resistance and poor patient treatment outcomes. Despite advancements in tumor resection and combination therapy, there is an urgent need for research into the advanced stages of PDAC to develop effective therapeutic strategies (Pramanik et al. 2024).

Nanomedicine has gained significant attention in cancer therapy due to its ability to enhance drug absorption, permeability, site specificity, and controlled release. Nanosystems can bypass biological barriers, prevent early drug degradation, and improve cancer diagnosis and treatment. They can be passively targeted to tumors through the enhanced permeability and retention (EPR) effect or actively targeted using ligands to improve the targeting ability (Liu, Shao, et al. 2025). The following chapters will focus on the analysis of GEM‐ or DSF‐loaded lipid‐based nanosystems that are among the most biocompatible and biodegradable nanodelivery tools, and on the combination of different anticancer agents within the same nanocarrier with the aim of improving efficacy against pancreatic cancer.

### 5.2 Lipid‐Based Nanosystems for Improved Delivery of GEM and DSF as Single Agents

#### 5.2.1 GEM Encapsulating Liposomes

Many research groups have focused on studying nanosystems to encapsulate GEM in order to increase its stability, cellular uptake, and safely maximize drug efficacy and therapeutic index (Habib and Singh 2021).

Hagiwara's group developed the liposomal GEM formulation FF‐10832 with improved stability and longer circulation half‐life compared to the free drug. Its safety and efficacy are currently being assessed in ongoing clinical trials, which include not only PDAC disease but also other solid tumors (https://clinicaltrials.gov/search?term=FF‐10832) (Matsumoto et al. 2021, 2022).

Among the various strategies proposed to overcome GEM resistance with delivery nanocarriers, the possibility of formulating liposomes functionalized with an active targeting agent is worth consideration. This approach consists of decorating the surface of the nanovector with ligands, aptamers, or monoclonal antibodies that recognize specific receptors overexpressed on tumor cells. This allows the nanosystems to deliver GEM preferentially to the target site and achieve better uptake (Silli et al. 2024; Tang et al. 2019; Zheng et al. 2024). Interestingly, Wu's group developed hyaluronic acid (HA)‐functionalised and GEM‐loaded pH‐sensitive liposomes (HA‐pSL) to study the influence of the active targeting on the efficacy of the GEM into two pancreatic cell lines: the Mia‐Paca‐2, sensitive to the drug, and the Gr2000, which are GEM‐resistant cells. The HA grafting allowed the increase of intracellular GEM in both cell lines and a higher impairment of the cell viability in Gr2000 when compared to untargeted liposomes. In vivo, tumor weights of Mia‐Paca‐2 and Gr2000 xenografts were significantly lower in mice receiving the HA‐pSL treatment than those treated with the pSL. Though, the size of Gr2000 tumor was twice larger than that observed in the MIA PaCa‐2 model, confirming the aggressiveness of the GEM‐resistant pancreatic cancer and highlighting the limitations of nanocarriers to overcome completely drug resistance.

Zheng et al. first confirmed the overexpression of P‐selectin in patient‐derived cancer tissues when they chose to decorate the liposomes with fucoidan molecules able to target this transmembrane protein and therefore the PDAC (Zheng et al. 2024). In this study, the targeting approach is combined with the use of pH‐responsive liposomes meant to improve the GEM delivery performances. However, when they compared the in vitro alteration in cell viability and the in vivo inhibition of tumor growth caused by fucoidan‐targeted liposomes and untargeted liposomes, the targeting strategy does not appear to confer better performance (Zheng et al. 2024). This might be due to the burst release of the targeting moiety, which is only electrostatically complexed with the liposomes or the insufficient expression of P‐selectin on the chosen cancer cell model Capan‐1 (Iwamura et al. 1997). Both Tang's and Zheng's studies have shown the limitations of tumor‐targeted delivery and revealed the complexity of chemoresistance, which goes beyond the therapeutic resistance linked to drug transport.

New approaches combining GEM‐liposomal formulations with physical stimuli such as mild hyperthermia were developed to investigate the potential improvement of GEM efficacy (Aparicio‐Lopez et al. 2024). In this study, it is shown the successful release of the drug upon temperature increase, and the encapsulated GEM was as efficient as the free GEM in impairing cell viability. In a different study, by Tucci et al., the same GEM‐loaded thermosensitive liposomes showed improved release profiles in serum and in vivo pharmacokinetics (Tucci et al. 2019). When assessed in vivo on the KPC pancreatic cancer cell line inoculated into the mammary fat pad of C57BL/6 mice, the GEM‐loaded thermosensitive liposomes combined with ultrasound hyperthermia did not show a greater efficacy than the free GEM, and the study was discontinued due to the high toxicity, most probably due from the high concentration of GEM found in the intestinal tract (Tucci et al. 2019). Nevertheless, these studies show the new trends of combining physical approaches with drug delivery nanosystems, which could become extremely powerful when fully mastered. In fact, the use of hyperthermia via High‐Intensity Focused Ultrasound (HIFU) technology has already been combined with neoadjuvant chemotherapy in clinics, with increased overall survival and better tolerability when compared to chemotherapy alone (Fergadi et al. 2022). Given that hyperthermia produced by HIFU at low energies (< 55°C) increases cell membrane permeability, we can combine it with thermosensitive GEM‐loaded liposomes. As these liposomes are designed to release their content in response to the same stimulus, we can boost the cellular drug uptake and, as a result, enhance the GEM treatment's efficacy.

For several years now, a great deal of research has been devoted to the synthesis of lipophilic prodrugs of GEM to improve its encapsulation within the liposomes. Wang et al. bound a C13 chain to GEM, enabling the preparation of liposomes that remained stable for over 2 years at temperatures between 2°C and 10°C, with no leakage of the drug (Wang et al. 2024).

Kim et al. prepared GEM‐C16 lipid chain‐loaded liposomes fused with human PDAC‐derived extracellular vesicles. This platform showed the potential to be effective for chemotherapy in patients with PDAC (Kim et al. 2024).

Li et al. prepared GEM‐C16‐loaded liposomes in which the surface was modified by binding a linear propanediamine derivative to the phospholipid component of liposomes (Li et al. 2019).

Masetto et al. prepared GEM prodrugs with nitric oxide (NO)‐donor moieties to enhance the metabolic stability and lipophilicity, facilitating encapsulation in liposomes. The release of NO from GEM into cells induced anti‐tumor effects and the most effective treatment was identified when the NO‐donor diethylamine NONOate was attached to GEM and encapsulated into the liposomes (Masetto et al. 2020).

Bulanadi and colleagues synthesized GEM‐phytanyl and formed lipid nanoparticles (GEM‐LPNPs) with 1,2‐dimyristoyl‐sn‐glycero‐3‐phosphocholine and cholesterol. GEM‐LPNPs exhibited reduced toxicity compared to free GEM, likely attributable to the sustained release of the drug (Bulanadi et al. 2020).

From a pharmaceutical development point of view, all of the above‐mentioned examples of lipophilic GEM prodrugs introduce additional steps into the proceedings, which include the chemical synthesis, the purification along with the characterization and quality control of the resulting conjugate. This may be costly and may introduce further requirements from regulatory affairs if the new entity is considered distinct from the original GEM.

Notably, Dora et al. did not modify the GEM molecule; instead, they developed lipid nanoparticles incorporating a phospholipid complex of GEM (GEM‐NPs) (Dora et al. 2024). The characterization of GEM revealed that it existed in an amorphous state within the lipid matrix, which allowed a controlled release via diffusion, with an initial burst release followed by a sustained one. GEM‐NPs demonstrated notable cytotoxicity against PDAC cell lines (Dora et al. 2024). Table 1 includes further information on the lipid‐based nanosystems encapsulating GEM.

**TABLE 1:** Summary of lipid‐based nanosystems for GEM delivery. The employment of block letters indicates in vitro and ex vivo experiments, while the use of italics indicates in vivo experiments.

Therapeutic system	Loaded drug	Phospholipid composition (molar ratio)	Pancreatic tumor models in vitro/ex vivo and in vivo (in italic characters)	Remarks	References
Liposomes	GEM (FF‐10832)	CHOL/HSPC/N‐MPEG‐DSPE 4/15/1	GEM metabolism and transport assessed in fractions of the liver, small intestine, tumor homogenates and isolated hepatocytes *Human Capan‐1and BxPC‐3 xenografted subcutaneously; SUIT‐2 orthotopically administered in BALB/cAJcl‐nu/nu mice*	Liposome formulation enhanced tumor drug targeting and improved therapeutic outcomes. A PBPK model revealed the pivotal roles of liposome stability, tumor accumulation, and metabolic activation in determining therapeutic success.	(Matsumoto et al. 2021, 2022)
Fucoidan‐coated cationic liposomes	GEM	DPPC/DMPC/DOTAP 4/1/1	Capan‐1 (human) Panc‐1 (human) Human tissues from resectable pancreatic ductal adenocarcinoma	Fucoidan helped to stabilize the liposomes and then improved the drug delivery in the tumor, showing that optimized PEGylated liposomes can be a promise and effective drug delivery system.	(Silli et al. 2024; Zheng et al. 2024)
Hyaluronic acid (HA) functionalized pH‐sensitive liposomes	GEM	DOPE/CHEMS/DSPC/CHOL/DSPE‐mPEG2000 HA‐DOPE/CHEMS/DSPC/CHOL/DSPE‐mPEG2000 4/2/2/2/0.3	MiaPaCa‐2 (human) Gr2000 (Mia‐PaCa‐2 resistant) *MiaPaca‐2 and Gr2000 xenografted in NOD Scid mice*	HA pH‐sensitive liposomes showed improved cellular uptake and in vitro cytotoxicity. In vivo efficacy studies also showed that decorated liposomes only partially resensitized cancer cells to GEM therapy.	(Tang et al. 2019)
Thermosensitive liposomes	GEM	DPPC/DSPC/DSPC‐PEG2000 80/15/50	KPC (mouse) BXPC3 (human)	Only the KPC cell line exhibited a response to liposomal treatment that was consistent with the anticipated response of an ideal thermosensitive liposome formulation.	(Aparicio‐Lopez et al. 2024)
Thermosensitive liposomes	GEM‐copper complex	DPPC/Lyso‐PPC/mDSPE‐PEG2000 86/10/4 DPPC/Lyso‐SPC/mDSPE‐PEG2000 86/10/4 DPPC/MPPC/mDSPE‐PEG2000 89/7/4 DPPC/MSPC/mDSPE‐PEG2000 89/7/4 DPPC/DSPC/mDSPE‐PEG2000 80/15/5	KPC (mouse) *KPC xenografted in C57BL/6 mice*	The interaction between copper and GEM is imperative for achieving elevated GEM concentrations within the loading buffers. This resulted in passive loading of GEM at concentrations that are increased compared to those reported in the literature.	(Tucci et al. 2019)
Liposomes	GEM‐C13 diester prodrug	GEM‐C13/DMPC/CHOL/mDSPE‐PEG2000 1/3/0.45/0.75	AsPC‐1 (human metastatic) SU.86.86 (human) *BALB/c nude mice and BALB/c mice bearing AsPC‐1 subcutaneous xenografted tumors*	The uptake of liposomes by pancreatic cancer cells was observed to be more effective than the free drug. In vivo, liposomes demonstrated a prolonged plasma half‐life, increased tumor accumulation, and superior antitumor efficacy compared to free drug.	(Wang et al. 2024)
Liposomes	GEM‐C16 prodrug	DOPC/CHOL/mDSPE‐PEG2000 70:25:5	Panc‐1 (human) KPC (mouse)	The hybrid nanosystem had enhanced targeting ability, resulting in increased accumulation at the tumor site and the potential for overcoming GEM resistance.	(Kim et al. 2024)
Liposomes	GEM‐C16 prodrug	LipoidE80/CHOL/mDSPE‐PEG/DSPE‐PEG‐2N 3:1:0.8:0.2	BxPC3 (human) AR42J (rat) Panc02‐luc (luciferase‐tagged murine) *Female C57BL/6 mice bearing Panc02‐luc*	Liposomes demonstrated superior therapeutic outcomes in an orthotopic pancreatic cancer mouse model relative to unmodified liposomes.	(Li et al. 2019)
Liposomes	Oxide‐releasing GEM prodrug	DSPC/CHOL/mPEG‐DSPE 65:30:5	MiaPaCa‐2 (human) Panc‐1 (human)	In vitro, liposomal delivery of the drug led to an increase in the level of apoptosis in PDAC cells and increased the expression of pro‐apoptotic proteins in comparison with standard GEM treatment.	(Masetto et al. 2020)
Liposomes	GEM‐phytanyl conjugate prodrug	DMPC/CHOL 73.5/8.1	CFPAC‐1 (human) BxPC3 (human) MiaPaCa‐2 (human) *NOD/SCID mice bearing* CFPAC‐1 *xenografted tumors*	The in situ enzymatic conversion of the prodrug to GEM was achieved with a high degree of effectiveness, depending on the enzyme concentration. In vivo, these liposomes exhibited superior antitumor efficacy in comparison with free GEM.	(Bulanadi et al. 2020)
Lipid nanoparticles incorporating a phospholipid complex	Phospholipid complex of GEM (GEM‐PC)	GEM‐PC/TPGS (0.5%–1% of surfactant)	MiaPaCa‐2 (human) Panc‐1 (human) *PDAC was induced in Sprague–Dawley rats using a chemical (DMBA) carcinogenesis approach*	In vitro investigations revealed that liposomes containing GEM demonstrated enhanced stability, efficacy, and safety. These systems exhibited resistance to enzymatic degradation.	(Dora et al. 2024)

Abbreviations: CHOL, cholesterol; DMPC, 1, 2‐dimyristoyl‐sn‐glycero‐3‐phosphocholine; DOPC, 1,2‐dioleoly‐sn‐glycero‐2‐phosphocholine; DOTAP, 1,2‐dioleoyl‐3‐triethylammonium‐propane; DPPC, 1,2‐dipalmitoyl‐sn‐glycero‐3‐phosphocholine; DSPE‐PEG‐2N, N,N‐dimethyl‐1,3‐propanediamine conjugated to DSPE‐PEG; Gd‐DSPE, 1,2‐dipalmitoyl‐sn‐glycero‐3‐phospoethanolamine‐N‐diethylenetriaminepentaacetic acid (gadolinium salt); HSPC, hydrogenated soy phosphatidylcholine; Lyso‐PPC, 1‐palmitoyl‐2‐hydroxy‐sn‐glycero‐3‐phosphocholine; Lyso‐SPC, 1‐stearoyl‐2‐hydroxy‐sn‐glycero‐3‐phosphocholine; MPPC, 1‐myristoyl‐2‐palmitoyl‐sn‐glycero‐3‐phosphocholine; MSPC, 1‐myristoyl‐2‐stearoyl‐sn‐glycero‐3‐phosphocholine; N‐MPEG‐DSPE or DSPE‐mPEG2000, N‐(carbonyl‐methoxypolyethylene glycol 2000)‐1,2‐distearoyl‐sn‐glycero‐3‐phosphoethanolamine sodium salt.

#### 5.2.2 DSF Encapsulating Liposomes

The encapsulation of DSF in liposomes has shown promising results to treat various types of cancer (Farooq et al. 2019), however, the number of studies concerning pancreatic cancer is limited. Marengo et al. focused on the development of hyaluronic acid (HA)‐decorated liposomes, containing the DSF derivative diethyldithiocarbamate‐copper [Cu(DDC)_2_], to target the CD44 receptor, a marker of CSCs in PDAC. The characterization of the liposomes revealed high encapsulation efficiency, and cryo‐TEM analysis confirmed the presence of Cu(DDC)_2_ crystals in the aqueous core. The liposomes exhibited robust antiproliferative effects on CSCs from PDAC cell lines or patient‐derived CSCs, predominantly through mechanisms involving ROS (Marengo et al. 2019).

### 5.3 Lipid‐Based Nanosystems for Drugs Co‐Delivery to Improve PDAC Treatment Outcomes

Drug‐combined delivery in pancreatic cancer has many advantages, such as the potential to overcome drug resistance, reduce toxicity, and address the problem of inadequate drug accumulation in tumor tissue (Duan et al. 2023).

**FIGURE 2:**
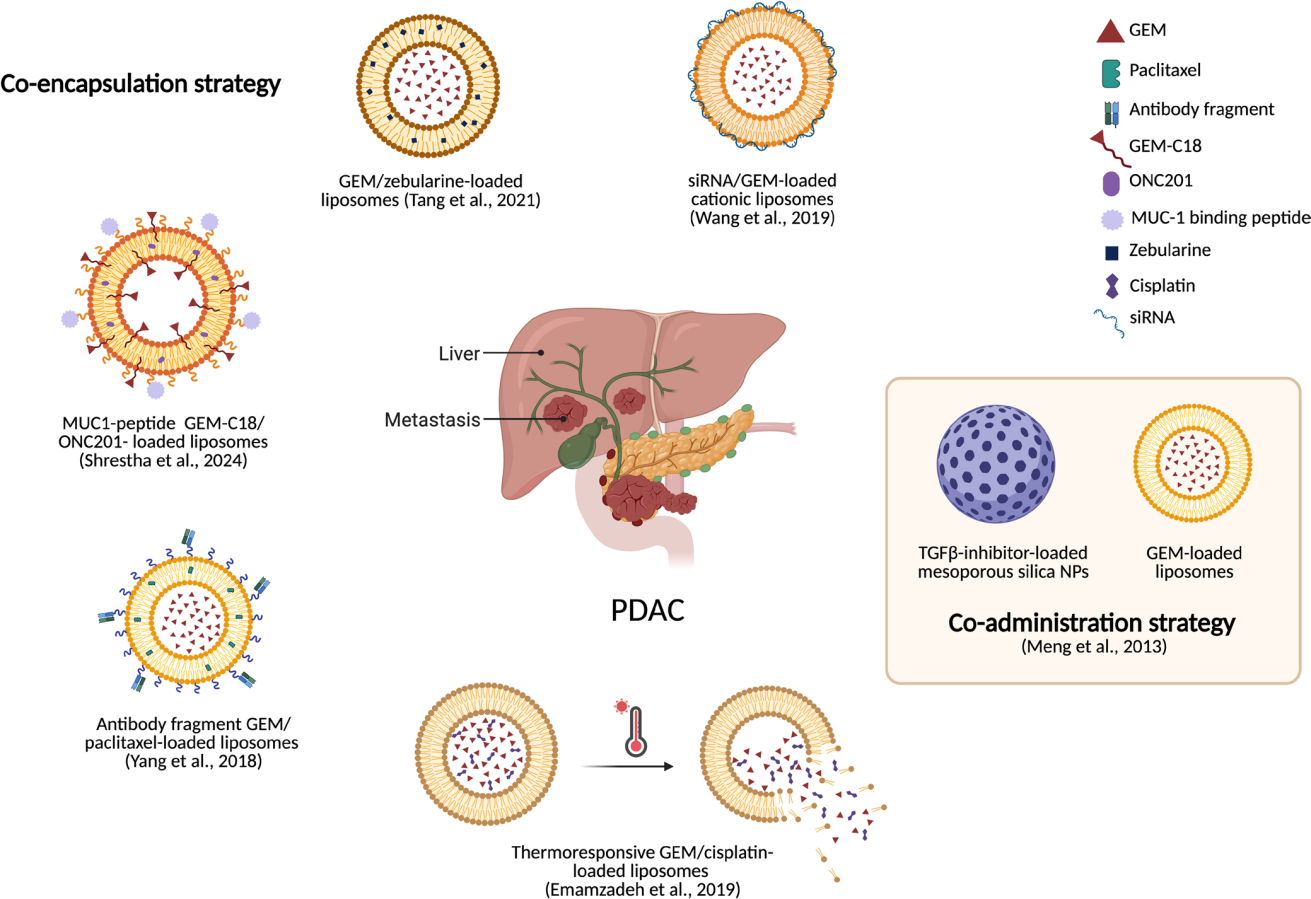
Examples of drug co‐delivery with GEM using liposomes (created with Biorender).

A considerable body of research has been conducted in recent years examining the potential of GEM in combination with other pharmaceutical agents within the same drug delivery nanosystem (Figure 2). This approach is meant to enhance the anticancer impact of GEM and decrease or delay the appearance of resistance to GEM (Miller et al. 2020; Nishimoto 2022).

Interestingly, Tang et al. tackled the therapy‐induced senescence correlated with GEM resistance by the use of a dual‐drug liposomal system (pSL‐GEM&ZEB) co‐encapsulating GEM and zebularine (Tang et al. 2021). The latter is an inhibitor of both DNA methyltransferase and cytidine deaminase (CDA), the enzyme responsible for GEM inactivation (Gesto et al. 2012). As the epigenetic modulators have been described as re‐sensitizers of drug‐resistant cells (Leary et al. 2018), the group emitted the hypothesis that the combination of zebularine with GEM would restore the efficacy of GEM in GEM‐resistant cells. The drugs were co‐delivered with pH‐sensitive liposomes (pSL), which provided an improvement in the GEM uptake and efficacy. Notably, the combined molecules zebularine and GEM abolished the appearance of the senescence phenotype, and morphological signs of apoptosis were observed upon 3 days of treatment compared to GEM alone. In vivo, liposomes reduced GEM clearance and prolonged its circulation time, increasing its bioavailability and half‐life. In rats, pSL‐GEM&ZEB showed a better pharmacokinetics profile than pSL‐GEM, corroborating the advantages of the co‐delivery (Tang et al. 2021).

With the same intent of counteracting GEM resistance, curcumin was co‐encapsulated with GEM into pH‐sensitive liposomes (PSL). Xu H. et al. examined the potential of curcumin to augment the efficacy of GEM in pancreatic cancer by impeding the activity of the MRP5 transporter, a primary contributor to GEM resistance (Xu, Li, et al. 2021). The GEM/curcumin co‐loaded liposomes increased GEM accumulation and cytotoxicity within Mia‐PaCa‐2 pancreatic cancer cells. In rats, PSL reduced plasma clearance of both drugs. This dual‐drug system offers enhanced drug delivery and efficacy, overcoming GEM resistance in vitro. Additional in vivo studies should follow, and further optimization is necessary for clinical use, in particular the amelioration of drug‐to‐lipid ratio in order to reach the dosage administered to patients (Xu, Li, et al. 2021).

The co‐encapsulation was also combined with the active targeting strategy. Yang W. et al. successfully developed an antibody fragment (AF) against the tissue‐factor (TF), conjugated to GEM/paclitaxel‐loaded liposomes (AF‐GPL), exhibiting enhanced uptake in pancreatic cancer cells compared to non‐targeted liposomes (GPL) (Yang et al. 2018). Unfortunately, this study did not verify the targeting specificity, since neither competition studies using the anti‐TF antibody nor comparisons with a TF‐negative cell line were conducted. Nevertheless, it was observed that the AF‐GPL cytotoxic effect was markedly increased in comparison to GPL. It is noteworthy that AF‐GPL induced apoptosis in a substantial number of treated cancer cells (Yang et al. 2018). The strategy of choosing the anti‐TF for targeting purposes could have positive outcomes not only for the increase of cytotoxic effects but also for the decrease of cancer‐associated thrombosis frequently observed in patients affected by pancreatic cancer (Khorana et al. 2007).

When looking for controlled drug release while maintaining synergistic effects of drug combinations, Emamzadeh et al. coated thermo‐sensitive liposomes (TTL) with a thermo‐responsive polymer to obtain polymer‐modified liposomes (PMTL) (Emamzadeh et al. 2019). The incorporation of both GEM and cisplatin (Cis) into PMTL led to a significant enhancement in the drugs' cytotoxicity in Mia‐PaCa‐2 and BxPC‐3 pancreatic cancer cells, when heated at 40°C (Emamzadeh et al. 2019). PMTL performed better than standard liposomes (TTL) due to its superior capability to release GEM and Cis at this temperature. Interestingly, the highest combination index, which indicates the degree of synergy between two drugs, was obtained when GEM and Cis were encapsulated into the PMTL formulation compared to GEM/Cis‐loaded TTL or free drugs. Overall, PMTL formulations enhanced drug delivery and cancer cell killing through a controlled, synergistic approach of multi‐drug therapies (Emamzadeh et al. 2019).

In a study, Wang Y. et al. investigated the combination of GEM chemotherapy and Mcl‐1 siRNA for the treatment of pancreatic cancer (Wang et al. 2019). The Mcl‐1 protein is overexpressed in pancreatic cancer cells, and its downregulation was found to enhance the effect of GEM. A liposomal formulation (LP‐Gem‐siMcl‐1) was developed to deliver both GEMs and the Mcl‐1 siRNA and enhance their uptake by tumor cells. The successful internalization of LP‐Gem‐siMcl‐1 by PANC‐1 and BxPC‐3 pancreatic cancer cells was accompanied by the silencing of Mcl‐1 and increased tumor cell death. In vitro, LP‐Gem‐siMcl‐1 demonstrated markedly enhanced efficacy, with a substantially higher reduction in cell viability compared to free GEM or siMcl‐1 alone. In mice, LP‐Gem‐siMcl‐1 was more effective than free GEM or siMcl‐1 at slowing tumor growth and led to increased tumor necrosis (Wang et al. 2019).

**FIGURE 3:**
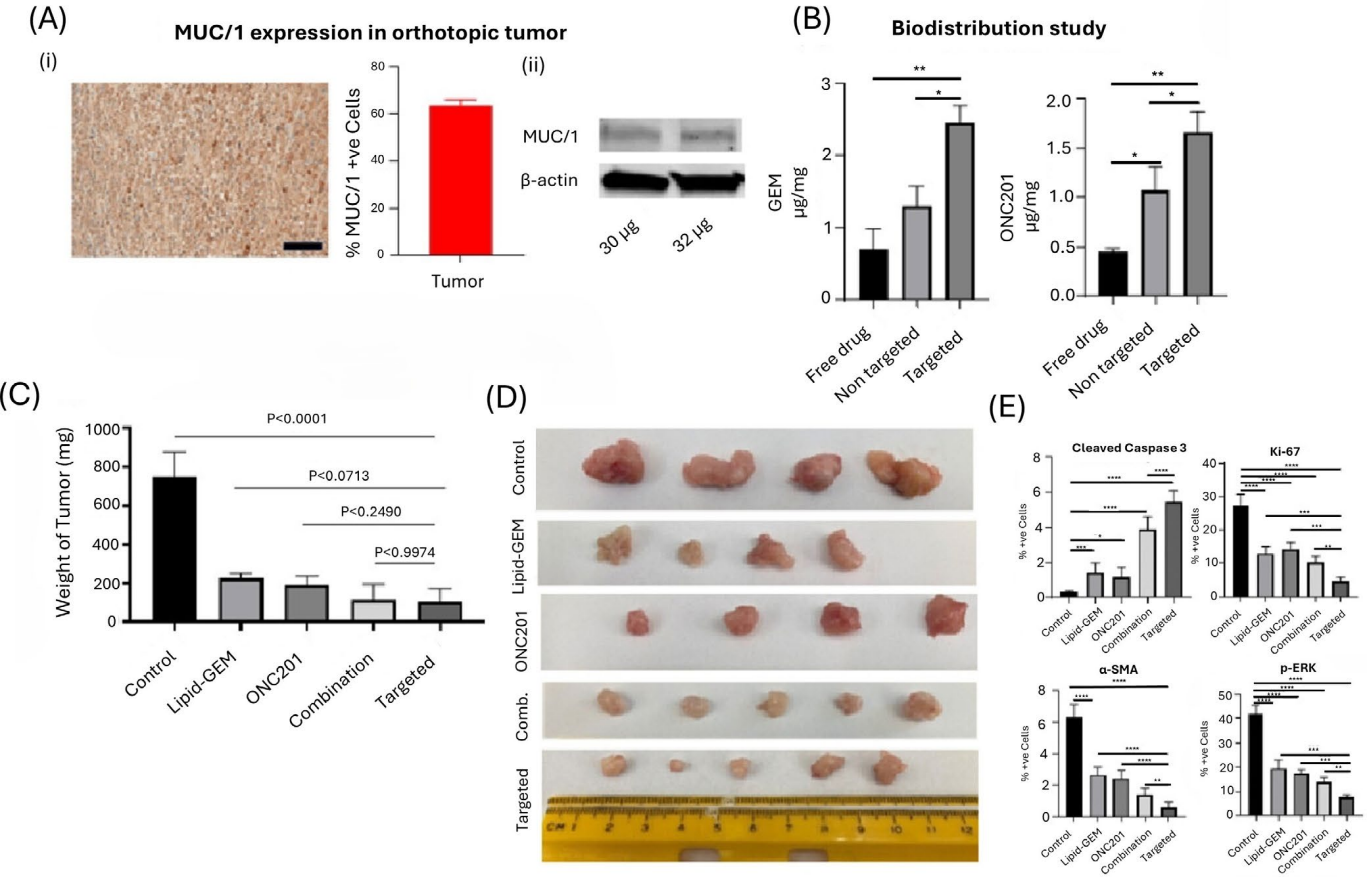
Adapted from (Shrestha et al. 2024). Biodistribution and therapeutic efficacy of untargeted or MUC‐1‐targeted liposomes loaded with the GEM–lipid conjugate, ONC201 individually, or their combination. (A) (i) Immunohistochemistry and (ii) Western blot analysis for assessing MUC1 expression in PDAC xenografted tumors. (B) Drug accumulation in the tumor at 6 h post systemic administration. (C) Weight of tumors. (D) Size of tumors. (E) Immunohistochemistry study of the expression of cleaved caspase‐3, Ki‐67, and α‐SMA and ERK proteins expressions in the excised tumors. Data presented as the mean ± SD (*n* = 5). *, **, ***, and **** represent *p* < 0.05, 0.01, 0.001, and 0.0001, respectively. Reproduced and adapted with copyright permission from ACS 2026.

Shrestha P. et al. conducted a comprehensive study in which several strategies were combined all together: the use of GEM lipophilic prodrug, the co‐encapsulation and the active targeting (Figure 3) (Shrestha et al. 2024). In this study, the authors investigated the potential of combining, within the same liposome, the stearoyl lipid‐GEM conjugate and the dordaviprone (ONC201), an inhibitor of AKT/ERK pathways and a potential candidate to resensitize cancer cells to GEM. This combination has been observed to enhance cell cycle arrest, induce ER stress, disrupt metabolic pathways and overcome GEM resistance by inhibiting the Akt/ERK pathways. The MUC‐1 high expression in orthotopic desmoplastic pancreatic tumor‐bearing NSG mice (Figure 3A) enabled the active targeting of these liposomes by means of the lipidic mucin 1 (MUC1)‐binding peptide for selective drug delivery. As shown in Figure 3B, the MUC‐1 targeted liposomes allowed an increased drug accumulation in the tumors compared to untargeted liposomes and the free drugs. In vivo studies also demonstrated that, despite targeted and untargeted lipid‐GEM‐ONC201 liposomes reducing tumor growth and size (Figure 3C,D), the MUC‐1‐targeted liposomes provoked the highest induction of caspase‐dependent apoptosis, as well as a reduction in the proliferation marker Ki67 and the metastasis marker α‐SMA (Figure 3E). Therefore, the targeted delivery of liposomes resulted in a more effective reduction in the aggressiveness and metastatic potential of PDAC (Shrestha et al. 2024).

In addition to combining drugs within a single nanosystem, another effective strategy involves combining several nanocarriers loaded with different drugs as part of a stepwise approach, in which the first drug‐delivery nanosystem reduces the biological barriers that might limit the efficacy of the second. Meng H. et al. combined two distinct types of nanoparticles: TGFβ inhibitor (LY364947)‐loaded mesoporous silica nanoparticles (TGFβi‐MSNP) and GEM‐encapsulating liposomes (Meng et al. 2013). The TGFβi‐MSNP demonstrated efficacy in targeting the tumor stroma, reducing stromal cell coverage of blood vessels, enhancing vascular permeability, and improving drug delivery. GEM was encapsulated in the liposomes to reduce toxicity and increase drug efficacy. The carriers were PEGylated to prolong the circulation time, and LY364947 was efficiently loaded onto the MSNPs to interfere with TGF‐β signaling. This approach demonstrated the potential to enhance drug delivery and immune responses by modifying the tumor microenvironment (Meng et al. 2013).

Kim et al. examined the potential of cromolyn, an S100P inhibitor, as a therapeutic agent for PDAC treatment (Kim et al. 2012). S100P overexpression is a common occurrence in pancreatic cancers, making it an ideal target for therapeutic intervention. Cromolyn was encapsulated in PEGylated liposomes (PEG‐lipo‐cro), resulting in an improved drug delivery system with enhanced anti‐cancer effects on S100P‐expressing BxPC‐3 pancreatic cancer cells. The inhibitory effect of cromolyn is further enhanced when combined with GEM‐loaded liposomes. In vivo, the PEG‐lipo‐cro formulation outperformed free cromolyn in inhibiting tumor growth and, when combined with PEG‐lipo‐GEM, showed enhanced effects without significant side effects, suggesting a promising cancer treatment strategy (Kim et al. 2012).

Despite the significant progress in the development of lipid‐based nanocarriers for the treatment of cancer, a number of inherent limitations still hinder their overall performance and clinical translation. Traditional nanosystems often suffer from instability, reproducibility, premature leakage of the drug, and limited regulation of release kinetics. This can lead to reduced therapeutic effect and increased systemic toxicity. Passive drug accumulation within tumors is also largely dependent on the EPR effect, which is prone to great interindividual variations and often proves to be insufficient when solid tumors present a stiff stroma and reduced vascularization such as the PDAC. Many current formulations fail to penetrate deeply into tumor tissues or to ensure localized and sustained drug release. Active targeting strategies, relying on surface modification with ligands, antibodies, or polymers, have provided some improvement in efficacy but still face significant hurdles regarding receptor heterogeneity, off‐target uptake, and often complex and expensive manufacturing processes. Overall, these issues highlight the need to develop innovative nanocarriers that are not only capable of ensuring structural stability, controlled release and optimal penetration, but are also compatible with biological systems and can be produced on a large scale.

**Sidebar title: External stimuli‐responsive nanomaterials**

Stimuli‐responsive nanomaterials offer targeted delivery by releasing therapeutic agents in response to specific external triggers such as light, ultrasound and magnetic fields. These smart nanomaterials have been engineered to improve treatment precision and efficacy, by enabling controlled and localized drug release (Elsherbeny et al. 2024).

The use of ultrasound‐responsive drug delivery is a promising approach for treating pancreatic cancer. This method allows for precise, image‐guided therapy and reduced adverse effects (Xia et al. 2016). Techniques like focused ultrasound (Gray et al. 2022) and microbubble‐assisted delivery (Jang et al. 2018) help drugs penetrate dense tissue, improving tumor treatment. Smart nanoparticles enhance effectiveness by releasing drugs in response to ultrasound or tumor‐specific conditions (Gao et al. 2019; Xing et al. 2016).

Photo‐responsive nanoparticles have emerged as a potentially efficacious therapy for pancreatic cancer, owing to their non‐invasive nature and the capacity for light‐triggered activation (Agiba et al. 2024).

The application of near‐infrared (NIR) light results in the generation of heat or reactive oxygen species, leading to cell death in tumoral cells (Lee et al. 2020; Mosaddad et al. 2023). Recent studies have demonstrated that the combination of NIR‐responsive nanoparticles and chemotherapy agents results in enhanced drug delivery, tumor targeting, and anticancer effects. While the preliminary findings are promising, the limited penetration of light in deep tissues poses a significant challenge for clinical implementation (Banstola et al. 2019; Ou et al. 2022).

Magnetic nanoparticles are effective at guiding drugs to tumors in the pancreas using external magnetic fields, reducing negative side effects and improving delivery of drugs (Moloney et al. 2023; Strobel et al. 2019). They are also used in magnetic hyperthermia, a process that uses heat to destroy cancer cells (Das et al. 2019; Lafuente‐Gómez et al. 2021). Research has shown that they can enhance treatment, especially with chemotherapy, but challenges must first be overcome before they can be used clinically (Oltolina et al. 2023; Socoliuc et al. 2020).

### 5.4 Criteria for an Ideal Nanodelivery System for GEM/DSF Combination and PDAC Treatment

The use of nanosystems has emerged as a strategy for delivering conventional drugs. A variety of nanocarriers have been formulated for combinational therapy, including polymer nanoparticles (NPs) (Paris et al. 2020), liposomes (Reiten et al. 2025), polymersomes (Shahriari et al. 2021), polymers/dendrimers (Song et al. 2020), carbon nanotubes (Tian et al. 2023), magnetic NPs (Chen et al. 2023), and mesoporous silica (Nasri et al. 2023). As previously discussed, GEM is a hydrophilic drug, while DSF is a hydrophobic drug. To encapsulate both drugs in a single nanosystem, it is necessary to employ a nanocarrier composed of two distinct compartments, each exhibiting higher compatibility with one of the drugs (Beutel and Halbrook 2023). Furthermore, as previous studies have shown that the increase of ROS production by DSF could promote the effect of GEM on pancreatic cancer cells (Dalla Pozza et al. 2011), it therefore is reasonable to propose that the administration of the two drugs should be sequential, starting first with DSF and then continuing with GEM. Liposomes are one of the most suitable delivery systems to achieve this sequential release, as DSF would be primarily encapsulated in the lipophilic bilayer of the nanocarrier and therefore released quickly, while GEM, due to its hydrophilicity would be loaded in the aqueous core of the liposomes, and it would be released with a slower kinetics.

**FIGURE 4:**
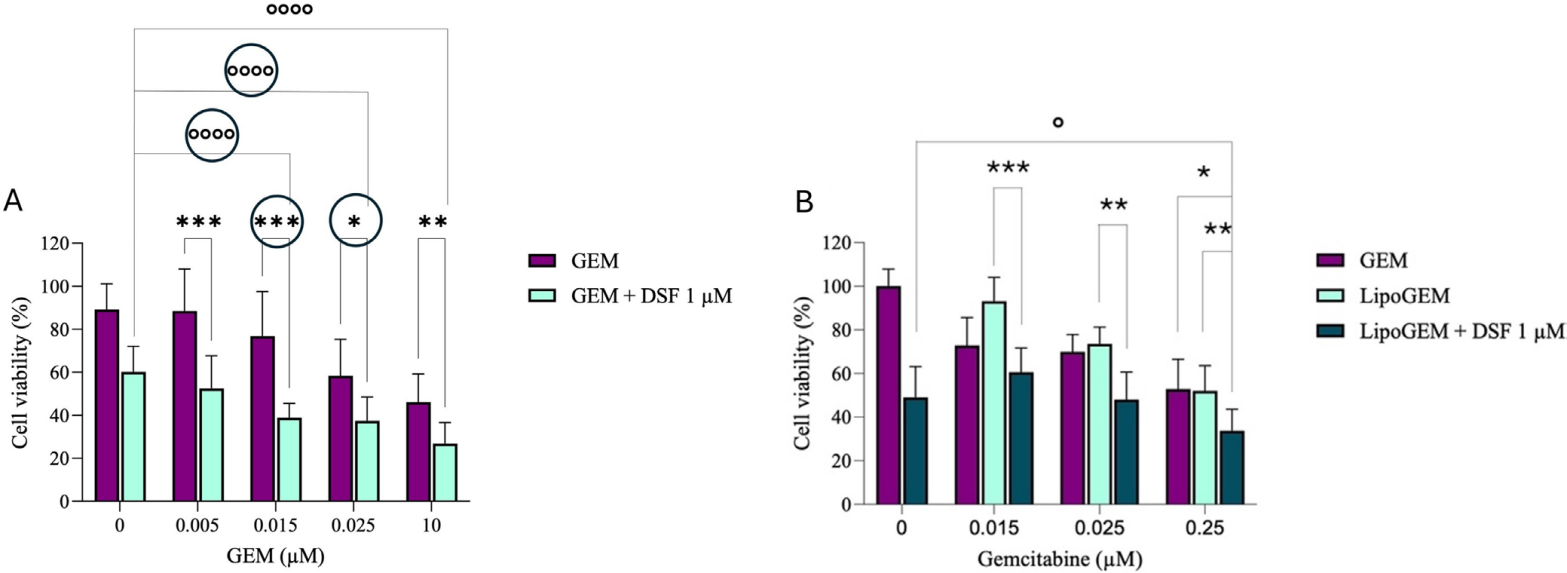
Adapted from (Bincoletto et al. 2026). Cell viability on PANC‐1 cells when treated with GEM (A), Lipo‐GEM (B) combined with DSF at 1 μM for 72 h. *X* axis corresponds to the GEM concentrations. The values shown at 0 μM identify the dispersing medium of the molecules (purple bar) and the viability of cells treated with DSF alone (light blue bar). Data are presented as means ± SD (*n* = 3). The *, **, and *** represent *p* < 0.1, 0.05 and 0.01, respectively versus GEM alone. The ° and °°°° represent *p* < 0.1 and 0.001 respectively versus DSF alone. The circled (O) statistics are the synergetic and most relevant concentrations of GEM and DSF to be combined for therapeutic purposes.

Nevertheless, despite the co‐encapsulation being advantageous for several reasons, drug release kinetics for both drugs should be fully mastered in order to reach the efficient doses that are necessary for synergy. As a matter of fact, our team conducted a systematic comparison of GEM and DSF doses required for the achievement of synergy (Bincoletto et al. 2026). We first tested the combinations of the free drugs, followed by GEM‐loaded liposomes combined with free DSF with the aim of mimicking sequential release (Bincoletto et al. 2026). Synergy was observed at various concentrations of the combined free drugs, regardless of whether they were administered concomitantly (Figure 4A) or sequentially with a 6‐hour interval, wheter GEM was added first followed by DSF or *vice‐versa* (data not shown). However, when GEM‐loaded liposomes were combined with free DSF, the synergistic effect was less pronounced. We anticipate that this is due to a slow release of GEM, preventing the system from reaching the effective doses needed to achieve synergy (Figure 4B).

Hence, in order to reproduce the synergistic dose–response effects observed with the free drugs, the multi‐drug nanosystems must take into account the fundamental parameter which is the release kinetics of each drug individually.

Other types of nanosystems that can be exploited include hyalurosomes, which are composed of phospholipids and HA (Zewail et al. 2025). Hyalurosomes can be exploited to encapsulate two drugs: in this case, lipophilic drugs are encapsulated in the phospholipid layer, and the hydrophilic therapeutic agents are entrapped in the aqueous core of the system. In this recent study, luteolin (LUT) and dexamethasone (DEX) were co‐encapsulated into hyalurosomes for the transdermal treatment of rheumatoid arthritis (RA). The co‐loading of these two active molecules within a single carrier enabled synergistic anti‐inflammatory effects while minimizing systemic exposure. The dual encapsulation strategy resulted in enhanced drug retention, sustained release, and targeted delivery to inflamed tissues. Notably, in vivo results showed that the co‐encapsulated system outperformed individual formulations in reducing joint inflammation and oxidative stress, offering a promising non‐invasive alternative to conventional RA injections (Zewail et al. 2025).

Another study investigated the co‐encapsulation of paclitaxel (PTX) and 5‐fluorouracil (5‐FU) into folic acid‐modified, lipid‐coated hollow mesoporous silica nanoparticles, which enabled the simultaneous delivery of both hydrophobic and hydrophilic drugs (Yin et al. 2022). In biological studies, the efficacy of this dual‐loading strategy was demonstrated to be superior to single‐drug‐loaded or free drug treatments in MCF‐7 breast cancer cells, with increased apoptosis and cell growth inhibition (Yin et al. 2022).

Polymersomes, similar in structure to liposomes but synthesized from a polymer instead of a lipid component, can encapsulate hydrophilic molecules in their aqueous interior and hydrophobic molecules in their membrane. Depending on the properties of the polymer or the encapsulated molecule, the drug release of polymersomes can be manipulated by pH, light, magnetic field, and temperature (Zhou et al. 2021).

Overall, among various nanocarriers that have been formulated as drug delivery systems, liposomes are one of those most widely utilized platforms. This is explainable by factors such as inherent biocompatibility, absence of immunogenic properties, along with their similarity to biological membranes, making them safer than various polymers or inorganic materials. Liposome‐based drug delivery systems are inherently divided into two compartments, with one compartment consisting of a water phase, while the other consists of a lipid bilayer. Therefore, liposomes are inherently suited for dual‐phase delivery of both hydrophilic as well as lipophilic compounds. Furthermore, liposome‐based drug delivery systems have a well‐established clinical track record, along with easy processability, since liposome composition could be tailored as per requirements of pharmacokinetics or targeting. There are various liposome‐based drug delivery systems that are FDA‐ as well as EMA‐approved, ensuring that liposomes are safe. Therefore, there are numerous reasons why liposomes are promising nanocarriers, among others, as future drug delivery platforms. Nevertheless, to design an efficient liposome specifically for the treatment of PDAC, one should carefully consider two aspects of this disease: the tumor microenvironment and the tumor immunosuppression (Malla et al. 2025). The ideal nanocarrier should be multifunctional to address each of these aspects.

The stiffness of the pancreatic stroma renders the delivery of the chemotherapeutics very challenging; therefore, it is fundamental to provide the disruption of the desmoplastic barrier to improve the response to chemotherapy, as previously described (Meng et al. 2013). This can be done by co‐encapsulating or co‐delivering modulators of the dense stromal component such as anti‐miR‐210 to inactivate stroma production (Xie et al. 2020).

Importantly, pancreatic tumors are also known for their ability to evade the immune system, a characteristic that contributes to the poor response to chemotherapy (Malla et al. 2025). Surface modification of liposomes with PD‐L1 antibodies or single‐chain fragments (scFv) may promote the infiltration by CD8+ T cells and the remodeling of the immune microenvironment (Tang et al. 2025), rendering the nanosystem a double‐edged sword.

## 6 Conclusion

Treatment options for PDAC have been stagnant for many years until the last decade, since when a better understanding of the complex biology of PDAC and the development of innovative therapeutic strategies have transformed the therapeutic landscape. Nevertheless, there are still persistent challenges ahead, particularly in terms of earlier diagnosis and overcoming chemoresistance. Recent preclinical promising results offer hope for more effective treatments. For instance, the combination of GEM with other drugs, such as DSF, has been shown to enhance antitumor effects and overcome resistance mechanisms. The synergistic potential of these combinations, particularly when paired with nanomedicine, represents a cutting‐edge therapeutic approach.

In fact, nowadays, the development of multi‐drug delivery systems for simultaneous delivery of multiple therapeutic agents is a key focus in nanotechnology‐based pharmacology. These systems use several compartments to encapsulate drugs with different physicochemical properties, enabling release rates to be controlled and mitigating adverse effects. In fact, if the drugs work synergistically, therapeutic doses can be reduced, resulting in less toxicity for patients. Nevertheless, the inability to precisely manage the ratio of loaded drugs, the difficulty in regulating release mechanisms, and the lack of targeting are limitations to be addressed. The integration of advanced drug delivery systems such as multifunctional nanocarriers will tackle these limitations and provide better treatment outcomes in PDAC. Despite the high potential of these multifunctional nanosystems, reproducibility, scalability, and standardization remain the main bottlenecks for their translation into the clinic. In fact, the manufacturing methods for most of these systems are not automatized (or this is not described in the articles), and heterogeneities often arise due to operator variability. The development of technologies such as microfluidics introduces constant and continuous processes that provide standardization and scalability at the same time. Comparative studies of microfluidic methods and traditional manufacturing processes showed ameliorated physicochemical properties of nanoparticles when produced by microfluidics, which ultimately affects the efficacy of these nanosystems (Jaradat et al. 2021; Rebollo et al. 2022).

Finally, and more generally, the new therapeutic strategies for pancreatic cancer should consider the health conditions of the patients to provide alternatives to the existing therapeutic regimens, which are not always tolerated. Particularly, cachexia affects up to 80% of patients affected by pancreatic cancer and this condition is often associated with a decreased treatment tolerance (Poulia et al. 2020; Talbert et al. 2019). New multi‐drug combinations and co‐encapsulating drug delivery nanosystems should systematically introduce not only efficacy studies but also toxicological and tolerance studies to understand how concretely these combinations can reach the clinic with real benefits for the patients.

Over the coming years, continued research and clinical trials will be essential to translate these promising strategies into new prospects for the fight against this aggressive disease.

## Author Contributions

**Valeria Bincoletto:** conceptualization (equal), writing – original draft (lead), writing – review and editing (equal). **Ilaria Andreana:** conceptualization (supporting), visualization (lead), writing – original draft (supporting), writing – review and editing (supporting). **Barbara Stella:** conceptualization (equal), supervision (supporting), validation (supporting), writing – review and editing (supporting). **Nazanine Modjtahedi:** conceptualization (equal), writing – review and editing (supporting). **Silvia Arpicco:** conceptualization (equal), supervision (equal), validation (equal), writing – review and editing (supporting). **Giorgia Urbinati:** conceptualization (equal), supervision (equal), writing – original draft (supporting), writing – review and editing (lead).

## Funding

The authors have nothing to report.

## Conflicts of Interest

The authors declare no conflicts of interest.

## Related WIREs Articles

Recent advances in sonodynamic therapy strategies for pancreatic cancer.

Theranostic nanoparticles for detection and treatment of pancreatic cancer.

## Data Availability

Data sharing is not applicable to this article as no new data were created or analyzed in this study.
